# Treatment of right hepatic artery stump bleeding after pylorus-preserving pancreaticoduodenectomy by covered stent endoprosthesis placement

**DOI:** 10.1016/j.radcr.2023.01.023

**Published:** 2023-02-02

**Authors:** Elif Can, Severin Daum, Julian Lenk, Sophia Paparoditis, Clarissa Hosse, Aboelyazid Elkilany, Maximilian de Bucourt

**Affiliations:** a Department of Diagnostic and Interventional Radiology, Charité-Universitätsmedizin Berlin, Hindenburgdamm 30, 12200, Berlin, Germany; b Charité - Universitätsmedizin Berlin, Corporate Member of Freie Universität Berlin and Humboldt-Universität zu Berlin, Department for Medicine (Gastroenterology, Infectious Diseases, Rheumatology), Hindenburgdamm 30, 12200, Berlin, Germany

**Keywords:** Arterial Intervention, Stent endoprosthesis, Hemorrhage, Pylorus-preserving pancreaticoduodenectomy, Hepatic artery

## Abstract

Serious complications after pancreaticoduodenectomy include rupture of pseudoaneurysms arising from pancreatic fistula and pancreatojejunostomy leakage. We report a case of successful endovascular minimally invasive treatment using a covered stent endoprosthesis of a right hepatic artery stump bleeding following pylorus-preserving pancreaticoduodenectomy that was not suitable for coil or glue embolization due to an insufficiently short neck.

## Introduction

Advances in surgical techniques and critical care have reduced perioperative mortality of patients undergoing pancreatic surgery up to 5% [1]. However, morbidity after pancreatic surgery remains high, reaching 30%-50% even in specialized centers [2,3]. Postpancreatectomy hemorrhage (PPH) is one of the most serious complications, occurring in 1%-8% of all pancreatic resections and accounting for 11%-38% of all surgery-related deaths [4,5]. Early PPH (within 24 hours) usually originates from a nonsecured vessel and is conventionally treated by re-laparotomy, while the best management of delayed PPH (after 24 hours) remains controversial. Delayed PPH usually originates from a pseudoaneurysm of a main branch of the celiac arteries or superior mesenteric artery (SMA) which develops secondary to a postoperative pancreatic fistula. Transarterial embolization has been advocated as a minimally invasive option to manage PPH, particularly when bleeding is associated with a pseudoaneurysm [6,7]. It is especially feasible when only minor branches need to be embolized. Some authors argue that the liver may even tolerate embolization of the common hepatic artery (CHA) without major consequences because blood supply is ensured via collaterals, such as the inferior subphrenic artery [7], [8], [9], in addition to the portal venous hepatic perfusion. However, patients undergoing pancreatoduodenectomy with lymphadenectomy may be left with less collaterals.

In such cases, embolization of the CHA may result in liver abscess, organ failure, or impaired healing of the hepaticojejunostomy, even if portal blood flow is maintained [9]. Several authors have reported placement of endovascular covered stents for the treatment of arterial pseudoaneurysm [8], [9], [10]. We report a case of successful treatment of a subtle but relevant bleeding from a proximally occluded right hepatic artery (RHA): Initially, the bleeding was repeatedly neither detected by high-end state-of-the-art computed tomography (CT) nor capsule endoscopy, but finally by angiography. A covered stent endoprosthesis extending from the proper hepatic artery into the left hepatic artery (LHA) could be inserted to exclude the bleeding because of sound collateralization from LHA into RHA.

## Case report

**Fig. 1:**
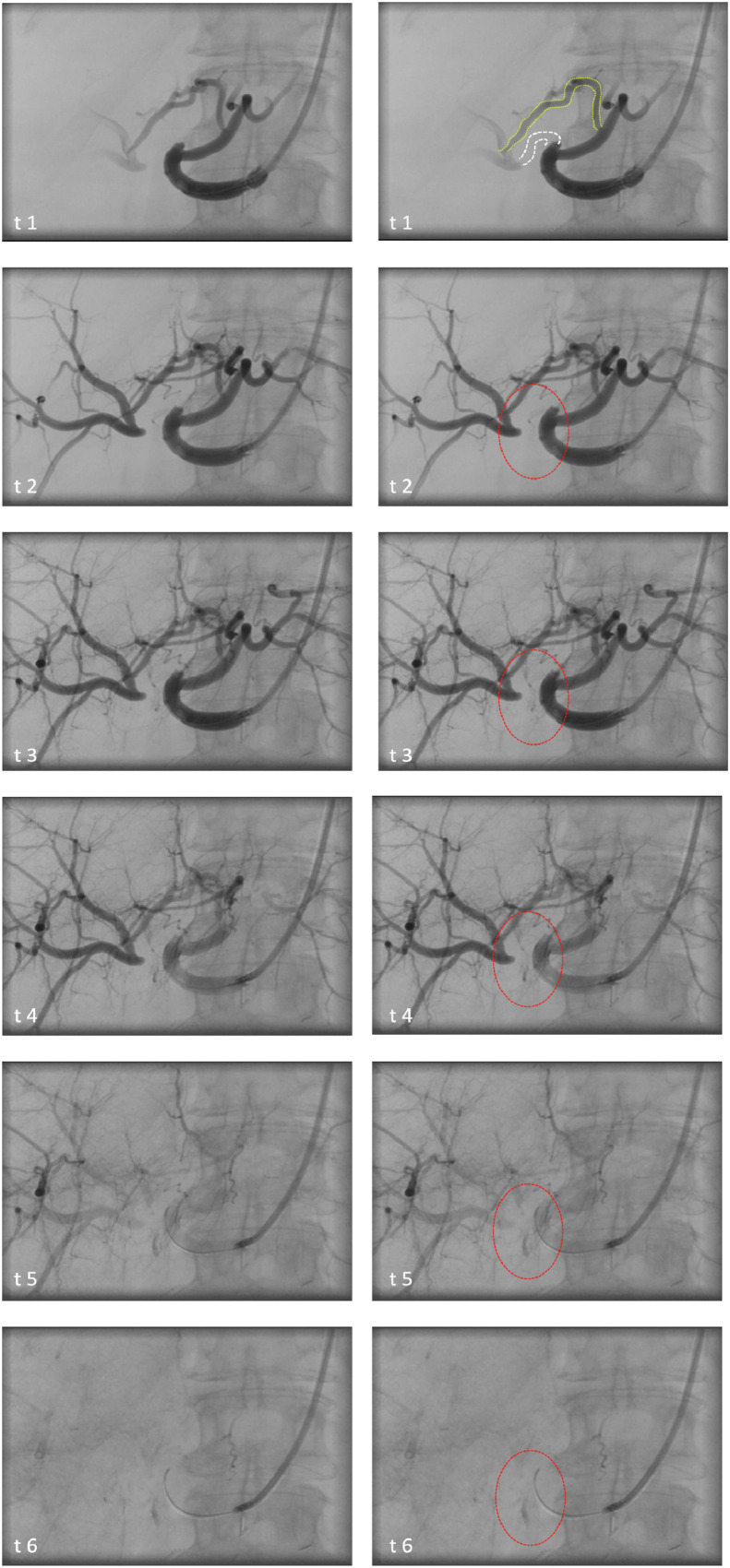
Left column: Angiography of proper, left and right hepatic artery (PHA, LHA, RHA) in time sequence t1-t6. Note that RHA is occluded proximally and reperfused distally via collaterals via LHA. Right column: Enhanced visualization with collateral flow from LHA to RHA (yellow dotted line in t1), proximally occluded RHA (white dotted lines in t1), and site of active bleeding increasing over time oval area encircled by red dotted line in t2-t6).

**Fig. 2:**
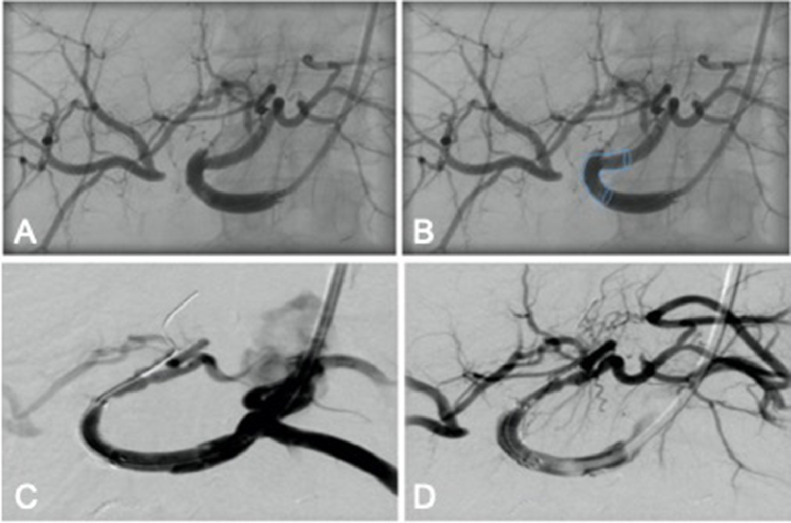
Angiography performed before stent placement (A and B) and after stent placement (C and D). Bleeding was managed via exclusion by placement of a covered stent endoprosthesis extending from the proper hepatic artery (PHA) into the left hepatic artery (LHA) while arterial perfusion of the right liver was preserved due to sound collateralization from LHA to RHA. A shows situation with proximally occluded and distally reperfused RHA via LHA collaterals; B enhanced visualization of planned covered stent location to exclude the bleeding. Control angiography (C and D) after covered stent placement shows patent lumen and collaterals to RHA.

A 79-year-old woman was referred to an external center with active gastrointestinal (GI) bleeding in the upper small intestine following pylorus-preserving pancreatic head resection with Y-Roux reconstruction 1 month before due to pancreatic head adenocarcinoma. The patient was re-hospitalized with suspected GI hemorrhage and hematochezia (hemoglobin of 6.2 g/dL). Endoscopy revealed ulcerative antral gastritis and mild anastomositis as possible sources of bleeding. CT angiography identified no active bleeding. Further episodes with hematochezia occurred requiring blood transfusion. Follow-up rectosigmoidoscopy and endoscopy with push-and-pull enteroscopy was performed. No active source of bleeding was detected. The patient was transferred to capsule endoscopy, which revealed active hemorrhage in the upper small intestine and double-balloon enteroscopy was performed. After transferral to the gastroenterology department in our hospital, the physical examination revealed no abnormalities apart from pale conjunctivae and a reduced turgor. Laboratory tests showed an increase in CRP to 59.8 mg/L and a moderate decrease in hemoglobin and liver enzymes compared to previous findings. Single-balloon jejunoscopy up to 240 cm postpylorus was performed and no bleeding source was identified. Low-molecular weight heparin was administered to carefully provoke and hopefully detect the source of bleeding. Another capsule endoscopy revealed ulcerous proximal jejunitis distal to the gastrojejunostomy, not sufficiently explaining the bleeding. In the following days several further Hb-relevant anal bleeding episodes occurred. Two emergency CT scans 6 and 7 weeks after surgery again failed to reveal the source of bleeding. Given the complex clinical workup with yet unsatisfactory results regarding the patient's clinical presentation, an abdominal angiography was performed, revealing small oozing hemorrhage from the RHA stump into the adjacent intestinal lumen (Fig. 1). Initially, especially given the short neck of the stump, a resorbable temporary local hemostyptic was carefully applied until the bleeding was no longer visible. Complementary SMA angiography revealed no additional bleeding sources and no arterial hepatic territory variants arising from the SMA. The intervention was discontinued and findings were discussed by general surgeons, gastroenterologists, and interventional radiologists to decide about further management in case of recurrent bleeding. When bleeding symptoms recurred, a second angiography was performed two days later (Fig. 1) and a covered stent endoprosthesis (5/25 mm Viabahn, Endoprosthesis, Gore) was successfully placed from proper hepatic artery into LHA to seal the source of bleeding by exclusion (Fig. 2; Videos). At the same time, arterial perfusion of the right liver was ensured via sound collaterals from LHA to RHA. Patency of the stent was confirmed at 5-month follow-up.

## Discussion

Traditionally, delayed PPH was addressed by direct surgical re-exploration which may be hazardous due to tissue friability. Also, bleeding site identification can be challenging. A recent meta-analysis comparing endovascular treatment and surgery revealed no significant differences between both treatments, although trends favoring endovascular treatment were evident in terms of complications (endovascular 36%; surgery 70%) and mortality (endovascular 21%; surgery 43%) rate [10]. Endovascular therapy is now considered the standard therapeutic approach for PPH since angiography may enable precise localization of the pseudoaneurysm [7], [8], [9], [10]. In some situations, placement of a covered stent endoprosthesis may be feasible, depending on individual vascular anatomy and branching patterns, which must ensure that sufficient hepatic arterial blood flow remains preserved. The increasing number of reports may reflect advances in therapeutic equipment and especially covered stent material. Depending on individual vascular anatomy (vessel diameter, tortuosity), it may be challenging or sometimes even impossible to reach the desired location for covered stent placement.

## Conclusion

In challenging clinical situations like the one presented here, an attentive and perseverant interdisciplinary team is needed to detect, understand, and possibly treat the underlying condition with a creative solution. The placement of covered stents with preservation of organ arterial flow, if technically possible, may in some cases represent the best or only treatment option, as in the case reported. Our patient had good collateralization from LHA to RHA but the RHA neck was too short for coil placement and too risky for glue embolization.

## Authors’ contributions

S.D., J.L., and M.d.B. performed the interventions. S.D. and J.L. were involved in medical care of the patient. E.C. wrote the manuscript; E.C. and M.d.B. prepared the figures. C.H., A.E., and S.P. were involved in manuscript writing and revision. All authors have read and approved the submitted manuscript.

## Availability of data and materials

All data generated or analyzed during this study are included in this published article.

## Patient consent

Written informed consent was obtained from the patient for publication of this case report and any accompanying images.

## Appendix Supplementary materials

Image, video 1

Image, video 2
